# Notices

**Published:** 1862-10

**Authors:** 


### NOTICES.

Friends' Review; weekly. Philadelphia. Three dollars a year. A Religious, Literary, and Miscellaneous Journal. Samuel Rhoads, editor—completing its fifteenth year, is giving a series of most valuable papers in reference to the early history of Maryland, in one of which it is stated that so early as a hundred years ago, one of the eighteen questions put to each individual at Friends' yearly meetings, was the following, number four: "Are all careful to keep their word, and pay their just debts and contracts in due time?" Who does not wish that the whole world was full of practical Friends, especially in this most important regard. "It would make a world worth living in," as wifey, at our elbow, this moment expressed herself. The biographical papers relating to David Cooper are worthy of being read and pondered on by every human being. He was great in his goodness (as Gurney, the personal friend of Legh Richmond, so known and revered by the "bishops and other clergy" of the Church of England, that they gathered around his funeral-bier, and testified, "Our brother is dead.") A right good stock for our children to hail directly from, and further improved by the sturdy Calvinism of their father.

Excursions.—Among the floating palaces of the Hudson, is Captain A. L. Anderson's new model steamer, the "Mary Powell," running up every afternoon some ninety miles, and returning by eleven o'clock next morning, enabling hundreds of our citizens to pass their nights on the banks of the glorious river, inhaling the delicious breezes, as they come up from the sea, away from the noise and dust and care and suffocating city heat. The successful enterprise of establishing this line of travel, so invaluable to multitudes of our citizens, conveys an instructive lesson to young men every where; for the Captain has not only accomplished a great public good, but has made for himself many friends, by a happy combination of business characteristics which, while in the reach of all who deserve success, are pretty sure to secure it in almost any department of legitimate business; indomitable energy, unswerving integrity, and firmness of purpose, joined with an accommodating courtesy. These are the qualities which have secured for Captain Anderson a comfortable fortune, and what is still more valuable to himself and children, a good name.

British Literature.—Leonard Scott and Co., No. 79 Fulton street, New-York, for ten dollars a year, furnish a reprint of the following Reviews: *London*, *Edinburgh*, *North British*, and *Westminster* quarterlies, with *Blackwood's* monthly. These publications are written for by the best minds and brightest intellects in Great Britain; they present to their readers a general view of the condition of the world as to literature, politics, and religion, and are well worthy of the patronage of liberal and educated men.

